# Retrospective evaluation of foot-and-mouth disease vaccine effectiveness in Turkey

**DOI:** 10.1016/j.vaccine.2014.01.071

**Published:** 2014-04-01

**Authors:** T.J.D. Knight-Jones, A.N. Bulut, S. Gubbins, K.D.C. Stärk, D.U. Pfeiffer, K.J. Sumption, D.J. Paton

**Affiliations:** a The Pirbright Institute, Pirbright, United Kingdom; b The Royal Veterinary College (VEEPH), University of London, United Kingdom; c The Şap Institute, Ankara, Turkey; d The European Commission for the Control of FMD, FAO, Rome, Italy

**Keywords:** Vaccine, Evaluation, FMD, Vaccine effectiveness, Turkey, Asia-1

## Abstract

Foot-and-mouth disease (FMD) is present in much of Turkey and its control is largely based on vaccination. The arrival of the FMD Asia-1 serotype in Turkey in 2011 caused particular concern, spreading rapidly westwards across the country towards the FMD free European Union. With no prior natural immunity, control of spread would rely heavily on vaccination.

Unlike human vaccines, field protection is rarely evaluated directly for FMD vaccines. Between September 2011 and July 2012 we performed four retrospective outbreak investigations to assess the vaccine effectiveness (VE) of FMD Asia-1 vaccines in Turkey. Vaccine effectiveness is defined as the reduction in risk in vaccinated compared to unvaccinated individuals with similar virus exposure in the field.

The four investigations included 12 villages and 1230 cattle >4 months of age. One investigation assessed the FMD Asia-1 Shamir vaccine, the other three evaluated the recently introduced FMD Asia-1 TUR 11 vaccine made using a field isolate of the FMD Asia-1 Sindh-08 lineage that had recently entered Turkey.

After adjustment for confounding, the TUR 11 vaccine provided moderate protection against both clinical disease VE = 69% [95% CI: 50%–81%] and infection VE = 63% [95% CI: 29%–81%]. However, protection was variable with some herds with high vaccine coverage still experiencing high disease incidence. Some of this variability will be the result of the variation in virus challenge and immunity that occurs under field conditions.

In the outbreak investigated there was no evidence that the Asia-1 Shamir vaccine provided adequate protection against clinical FMD with an incidence of 89% in single vaccinated cattle and 69% in those vaccinated two to five times.

Based on these effectiveness estimates, vaccination alone is unlikely to produce the high levels of herd immunity needed to control FMD without additional control measures.

## Highlights

• We assessed foot-and-mouth disease (FMD) Asia-1 vaccine effectiveness in Turkey.
• Retrospective cohort methods were used after village FMD outbreaks.
• For the vaccine containing the FMD Asia-1 Sindh-08 antigen: vaccine effectiveness = 69% against clinical disease and 63% against infection.
• The vaccine containing FMD Asia-1 Shamir antigen did not protect.

## 1 Introduction

Foot-and-mouth disease (FMD) vaccines are used on an enormous scale across the globe, with over 2 billion doses thought to be used every year [1]. Despite this, little is done to assess their performance in the field. Vaccine effectiveness, defined as the reduction in risk in vaccinated individuals compared to similarly exposed unvaccinated individuals under field conditions [2], provides a direct measure of vaccine protection within a vaccination programme.

**Fig. 1:**
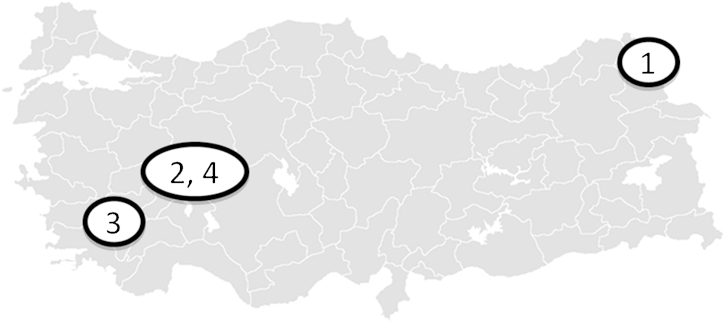
Map showing locations of foot-and-mouth disease outbreaks investigated. 1) Ardahan province, Turkey September 2011 (*n* = 296); 2) Afyon province, Turkey January 2012 (*n* = 218); 3) Denizli province, Turkey June 2012 (*n* = 405) and 4) Afyon province, Turkey July 2012 (*n* = 311). Investigation 1) looked at the FMD Asia-1 Shamir vaccine. Investigations 2–4) investigated the new FMD Asia-1 TUR 11 vaccine (Sindh-08 strain).

FMD in Anatolian Turkey (Fig. 1) poses a threat to the EU which is disease free [3]. During 2009–11 (inclusive) approximately twenty-million doses of polyvalent FMD vaccine were used a year for biannual mass vaccination of Turkey's cattle population [4]. In Turkey, inactivated, oil adjuvanted FMD vaccines with a specified protective effect of >3PD_50_ (PD_50_ = 50% protective dose) are administered intra-muscularly.

In 2011 Turkey experienced an incursion of the FMD Asia-1 serotype. Although serotypes A and O are endemic this serotype had not been present since 2002 [5]. Vaccine matching tests suggested that the vaccine used at the time (Asia-1 Shamir) would not protect against the new field strain (FMD Asia-1 Sindh-08) [6]. In light of this and the rapid spread of the Asia-1 virus across most of Turkey, the Şap institute, the Turkish FMD research institute and vaccine producer, changed their Asia-1 vaccine strain from Shamir to an isolate of the circulating Sindh-08 field virus (the vaccine is referred to as the TUR 11 vaccine). In this study, we investigated FMD Asia-1 vaccine effectiveness for both the TUR 11 and Shamir vaccine through retrospective outbreak investigations.

## 2 Materials and methods

Four retrospective outbreak investigations were conducted between September 2011 and July 2012. The investigations examined cattle in village small holdings. Suitable village outbreaks were identified from central records with the assistance of local veterinary services. Villages eligible for the study fulfilled the following criteria:

- A recent FMD Asia-1 outbreak had been reported.
- The outbreak had recently finished or was in the tail end of the epidemic. The course of an outbreak was determined from oral accounts from villagers and state veterinarians who visited the outbreaks repeatedly. An outbreak was deemed to be in the tail end if few or no new cases were detected the week before the investigation.
- The Asia-1 vaccines under investigation had been used in the village within the six months prior to the outbreak, i.e. trivalent FMD vaccine against serotypes A, O and either Asia-1 Shamir or Asia-1 TUR 11 produced by the Şap institute, Ankara, Turkey.

The outbreaks investigated were the only ones found at the time that fitted the criteria. Investigated villages also complied with the following:

- They had no history of prior exposure to FMD Asia-1.
- Farmers could recall which cattle were vaccinated (e.g. farmers had few cattle or a consistent vaccination routine).
- Farmers were aware of which animals developed FMD.

Details of the four investigations are presented in Table 1 and Fig. 1.

### 2.1 Sampling and data collection

Each investigation lasted for approximately eight days. Each village was visited by the investigation team (Knight-Jones and Bulut plus an assistant). Details of livestock management, vaccination and FMD history were gathered for the village. Then, households with known FMD virus exposure were sampled, i.e. those with cases or known contact with cases. If there was insufficient time to include all eligible households, equal proportions of households were selected from different geographic sections of the village. Within households, FMD vaccination and clinical history were collected for each animal. Animals were blood sampled and received an oral examination examining the hard palate, gums, lips and tongue (extruded) except when impossible or unsafe.

Oral vesicles and blisters typically appear about four days after infection. They typically heal within 10 days, leaving a scar that becomes less visible over time, although foci lacking lingual papillae may be visible for weeks [7]. As appearance of clinical signs is strongly correlated with shedding and transmission, this is a relevant outcome for assessing vaccine protection.

Full details of data collected are provided in table S1 (supplementary material).

### 2.2 Analysis

All analysis was done at the individual animal level unless stated otherwise. An animal was considered affected by FMD if detected on examination or seen by the farmer. All farmers were familiar with FMD. Vaccination status refers to whether an animal was vaccinated at the previous round of mass vaccination (done within the last six months). In the TUR 11 investigations, aside from the single round of vaccination with the trivalent A, O, Asia-1 TUR 11 vaccine, earlier FMD vaccination only included A and O strains. In the Shamir vaccine investigation (Ardahan province, Eastern Turkey), animals had been repeatedly vaccinated with the Asia-1 Shamir vaccine every six months. In this latter investigation FMD risk by number of doses received in an animal's life was also evaluated.

### 2.3 Validation

#### 2.3.1 FMD status

Farmer reported FMD status was compared to findings from clinical examination to assess the sensitivity and specificity of farmer detection. FMD status (farmer reported or detected on examination) was compared to NSP sero-status, since convalescent animals should be NSP sero-positive.

#### 2.3.2 Vaccination status

True vaccine status, as recorded by the government vaccinator at the time of vaccination was compared to farmer reported vaccination status. Government records were not available for all villages.

### 2.4 Vaccine effectiveness

To remove the effect of maternally-derived-immunity, all animals under five months were excluded from the analysis. Descriptive data analysis was performed. Crude vaccine effectiveness, *V*_*E*_, was calculated as:

(1) $$V_{E}=1-\frac{R_{V}}{R_{U}}$$

where *R*_*V*_ and *R*_*U*_ are the attack rates (percentage affected) in the vaccinated and unvaccinated populations, respectively. Univariable analysis of potential risk factors for clinical FMD was performed. As crude VE estimates, not adjusted for confounding, can be misleading, VE was calculated whilst adjusting for one factor at a time by stratification, see Table 2 with more detailed results in table S2 (a) and (b).

### 2.5 Regression modelling

To simultaneously adjust for several confounders, a multilevel, multivariable, binomial regression modelling was constructed using a complementary log–log link function. To account for the hierarchical structure of the data a random intercept was included, varying by village and management group nested within village. This class of model provides estimates of the log of the rate ratio [8] that can be used to determine VE using Eq. (1). Regression modelling was carried out in a Bayesian framework to allow for uncertainty in the time-at-risk for each animal.

### 2.6 Model building

A forward fitting approach was used adding vaccine status to the model first followed by the other exposures in order of decreasing univariable strength of association with the outcome. A factor was retained if it improved model fit or removed confounding. All two way interactions were investigated. Non-informative prior distributions were used (diffuse normal for regression coefficients and uniform for the standard deviation of random effects). Squared standardised deviance residuals were assessed and a global goodness-of-fit Bayesian *p*-value calculated using posterior predictive checking [9].

### 2.7 Time at risk

A time offset was included in the model representing time-at-risk, though this was not directly observed. To incorporate uncertainty in the time-at-risk, this parameter was sampled from a uniform distribution with minimum and maximum values as follows: for non-cases, the minimum was the number of days between the start of the village outbreak and the investigation and the maximum was the number of days between last vaccination and the investigation. For those with FMD, time at risk ended halfway between the time of the outbreak and the investigation for both the minimum and maximum. This represented the average time when a case would become affected.

### 2.8 Outcomes

The model was also used to estimate VE against severe disease, i.e. severe enough for an animal to stop eating or where oral lesions had a combined diameter of greater than 50% of the width of the hard palate (approximately).

VE against infection was calculated. An animal was considered infected during the outbreak if it tested positive for both NSP antibodies (>50% percentage inhibition, standard cut off) and Asia-1 structural protein (SP) antibodies (reciprocal titre >32, standard cut off), the former tested using the PrioCHECK FMDV NS ELISA (Prionics, Zurich, Switzerland) and the latter by titration with the Asia-1 Liquid Phase Blocking ELISA (The Pirbright Institute, UK). There is some uncertainty over the relative reactivity of the LPB ELISA, which uses the Asia-1 Shamir antigen, with cattle vaccinated with the Shamir vaccine and infected or vaccinated with the Sindh-08 strain. The possibility of low sensitivity due to differences in the field virus and the ELISA antigen provided a further reason for using the 1:32 titre cut-off and not higher. Testing was performed at the Şap institute, Ankara, Turkey.

The relationship between within-group incidence and within-group vaccine coverage was investigated.

Preliminary analysis was done in R [10] with the lme4 package [11], while the Bayesian analysis was implemented in OpenBUGS [12].

### 2.9 Vaccine match and batch release testing

Vaccine matching tests had previously been done at WrlFMD. *r*_1_-Values were 0.13–0.27 for the Shamir vaccine and >0.81 for the TUR 11 vaccine with the Sindh-08 field strain (an *r*_1_-value <0.3 suggests poor vaccine match) [6].

All vaccine batches are routinely tested to ensure that they elicit an “adequate” immune response. Tested at point of production in five cattle, 28 days after vaccination with a single dose, cattle had a mean virus neutralisation reciprocal titre of 24 for both vaccine batches used at Ardahan and Denizli, 29 for the batch used in Afyon-1 and 34 for the two batches used at Afyon-2 (assessed against vaccine homologous virus). The cut-off titre for protection found in challenge studies was 16 (as per OIE guidelines [13]).

Post-vaccination immune response was also assessed during the investigations in cattle not affected by or exposed to FMD.

## 3 Results

**Fig. 2:**
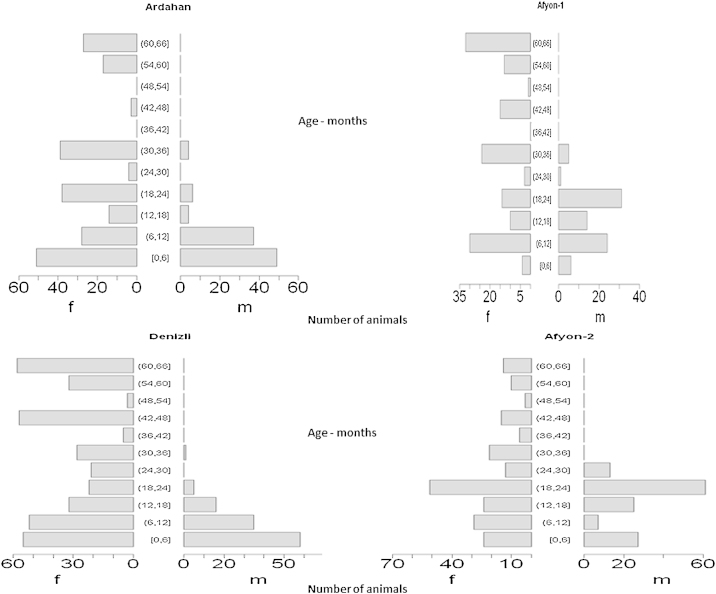
Population pyramids showing numbers of cattle sampled in the four investigations broken down by age and sex.

In total, 1377 cattle were included in the study of which 1230 were over four months of age. The cattle included in the four investigations were from 134 management groups from 97 different holdings in 12 villages. Typically, almost all households in a village would own some cattle (inter-quartile range 5–15 cattle per holding). See Fig. 2 for the age-sex structure.

Oral examination was performed on 82% (611/742) of cattle ≤24 months and 42% (207/488) of cattle >24 months of age. All (724/724) cattle ≤24 months were blood sampled and 99% (484/488) of those >24 months.

The percentage of cattle over four months old with clinical FMD ranged between 36% and 70%, depending on the investigation. The proportion infected (NSP positive with Asia-1 SP titre ≥32) was 86% in the unvaccinated (222/257), 65% in the TUR 11 vaccinated cattle (211/327) and 89% in the Shamir vaccinated cattle (129/145).

Vaccine coverage of animals over four months was 84% (Ardahan investigation), 42% (Afyon-1 investigation), 83% (Denizli investigation) and 60% (Afyon-2 investigation). The Shamir vaccine was only used in the Ardahan investigation except for eleven cattle in the Afyon-1 investigation.

Table 2 shows both descriptive statistics and univariable associations with clinical FMD with more details in table S2 (a) and (b). All factors except trimester of pregnancy (*p* = 0.3) showed some degree of association with clinical FMD (*p* < 0.1) (i.e. vaccination status, age, use of common grazing, breed, sex, herd size, time since vaccination, herd vaccine coverage and the investigation).

### 3.1 Validation

#### 3.1.1 FMD status

Of the 394 animals with clinical FMD on examination, farmers reported disease in 283 (detection sensitivity of 72%). This showed little variation with herd size (*p* = 0.1). Failure to detect FMD will result from mild disease or limited farmer observation and recall.

Cases where the farmer reported disease but clinical signs were not apparent on examination (47/371 [13%]) will result from recovery or recall error. The remaining 87% where both the farmer and the examination did not detect disease gives a pessimistic estimate of farmer specificity. Detection rates were similar for vaccinated and unvaccinated cattle (*p* = 0.6), so misdiagnosis should not bias vaccine effectiveness estimates.

#### 3.1.2 Vaccine status

Accurate government vaccine records were available for 372 animals. From these, 280/287 were correctly reported as vaccinated by the farmer (98% accuracy [95% CI = 95%–99%]). This error rate was unaffected by FMD status (*p* = 0.25). Farmer reporting was correct for 83/85 unvaccinated cattle (98% [95% CI = 92%–100%]). Again, FMD status did not affect this misclassification (*p* = 0.14). After exclusion of young calves, only one vaccinated and one unvaccinated animal were misclassified from 263 vaccinated and 57 unvaccinated cattle.

### 3.2 Multiple vaccine doses – Shamir only

After multiple doses of the Shamir vaccine, risk of FMD fell from 89% in single vaccinated cattle to 40% in those with more than five doses over their lifetime (see Table 3).

### 3.3 Crude VE

Crude estimates for VE are presented stratified by different variables (Table 2), according to different clinical outcomes (table S3) and for infection assessed by different serological criteria (table S4). However, due to confounding limited conclusions can be drawn from crude VE estimates (see regression model below).

VE varied with time between vaccination and the outbreak. For the TUR 11 vaccine VE appeared to decline markedly after 100 days (Table 2). For the Shamir vaccine, crude VE was 65% [95% CI: 22%–84%] if the outbreak occurred within 50 days of vaccination compared to VE = −54% [95% CI: −30% to −80%] and −14% [95% CI: −39% to 6%] if the outbreak occurred 51–100 days and 101–150 days after vaccination respectively. This effect could not be assessed in the multivariable analysis due to collinearity.

### 3.4 Regression model

Posterior median VE for the TUR 11 vaccine was 69% [95% credible interval (95% CI): 50%–81%]. No protective effect was detected for the Shamir vaccine (VE = −36% [95% CI: −140%–21%]) (Table 4). Against severe disease VE was 83% [95% CI: 67%–92%] for the TUR 11 vaccine. VE against infection was 63% [95% CI: 29%–81%] for the TUR 11 vaccine. Credible intervals were too wide to interpret the Shamir vaccine effect. Cattle from small herds (≤30 cattle) and cattle that used common grazing had a greater risk of FMD (Table 4).

Although there was no difference in squared standardised residuals in the four different investigations (*p* = 0.97), model fit did vary by village (*p* < 0.0001). Reasons for this were not apparent, but it may result from factors not included in the analysis that were more important in some villages than others or differences in data accuracy, which may differ by village.

### 3.5 Within-herd incidence and coverage

**Fig. 3:**
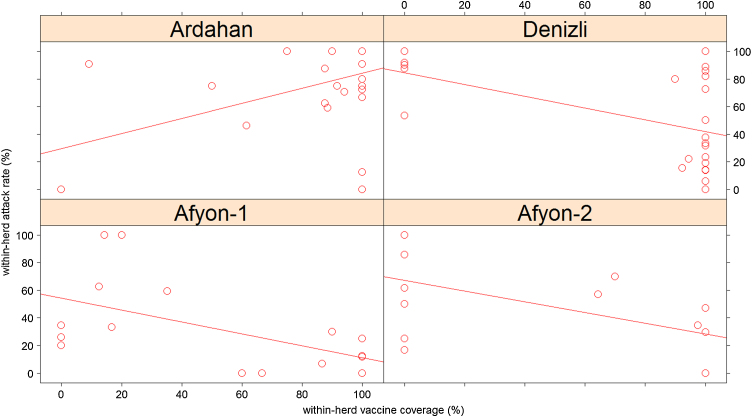
Relationship between within-herd vaccine coverage (i.e. the percentage of animals vaccinated with the FMD Asia-1 vaccine at the round of vaccination prior to the outbreak) and within-herd attack rate (i.e. the percentage of animals with clinical FMD during the outbreak) for cattle over four months of age for all households. The solid line is the best fit line to the data. The FMD Asia-1 TUR 11 vaccine was used in all outbreaks except in the Ardahan investigation where the FMD Asia-1 Shamir vaccine was used.

In the Afyon-1 and Afyon-2 investigations (TUR 11 vaccine), a within-herd incidence >50% only occurred in herds with <75% vaccine coverage. In the other TUR 11 study (Denizli province) although many of the high coverage herds had low incidence, high incidences (up to 100%) occurred in herds with 100% coverage. Outbreaks in unvaccinated herds always had high incidence (>50%). Unlike the Shamir investigation, in the TUR 11 investigations within-herd FMD incidence tended to decline with increasing vaccine coverage (Fig. 3).

In the Shamir investigation, cattle were at grass and group refers to large grazing groups (16 groups for 32 farms). In the TUR 11 investigations cattle were either permanently housed or housed at night.

### 3.6 Post-vaccination immune response

In the Afyon-1 investigation additional cattle were sampled from a nearby village that did not experience an outbreak but were vaccinated with the same vaccine batch at approximately the same time. These 50 sera had mean Asia-1 LPB ELISA titres of 119 (or 10^2.08^) for cattle less than seven months old, 153 (10^2.18^), 237 (10^2.37^) and 206 (10^2.31^) for cattle 7–12 months, 13–24 and over 24 months respectively. The proportion with an Asia-1 SP titre ≥100 (10^2^), a threshold associated with clinical protection, in the different age categories (in the same order) was 2/6 (33%), 9/17 (53%), 8/8 (100%) and 15/19 (79%) respectively.

In the outbreak villages, 27/29 (93%) of blood sampled cattle that were NSP negative and did not have clinical FMD had an SP LPBE titre ≥100.

## 4 Discussion

A single dose of FMD Asia-1 TUR 11 vaccine was effective at protecting against clinical disease, VE = 69%, particularly severe disease, VE = 83%. The vaccine also protected against infection, VE = 63%. The FMD Asia-1 Shamir vaccine did not appear to protect, indicated by (i) the vaccine effectiveness estimate, (ii) the high incidence in vaccinated cattle and (iii) no reduction in incidence until animals had received >5 doses of vaccine. However, findings for the Shamir vaccine are based on one investigation.

Although disease enhancement after vaccination has been identified for some other diseases the negative vaccine effectiveness for the Shamir vaccine is probably an artefact (residual age-confounding and collinearity). The confidence intervals show the uncertainty in the modelled Shamir VE.

It could be argued that outbreaks are cases of vaccine failure that do not represent typical vaccine performance. If so, vaccine effectiveness estimates would be pessimistic. That said, findings were consistent with (a) vaccine matching *r*_1_-values which suggested a good match for the homologous TUR 11 vaccine and a poor match for the Shamir vaccine (see Section 2) and (b) the large number of outbreaks seen within the Turkish vaccination programme. VE for the TUR 11 vaccine is comparable with the 60%–85% vaccine efficacy that would be expected for a 3PD_50_ vaccine [14] and is close to OIE batch release requirements where >70%–75% of vaccinated cattle must have a protective titre [13].

When comparing the Shamir and TUR 11 vaccines, differences in VE are consistent with differences in vaccine match *r*_1_-values. The closest we had to a direct comparison of the two vaccines was in Afyon-1 where 11 doses of Shamir vaccine were used in one village whilst TUR 11 vaccine was used in the other investigated village. The TUR 11 vaccine was approximately twice as effective with 3/11 (27%) affected in cattle vaccinated with the Shamir vaccine and 11/80 (14%) in the TUR 11 vaccinated cattle (see Table 2), however, this comparison was under-powered.

TUR 11 vaccine performance varied, possibly due to variability in (1) field conditions, e.g. season, time since vaccination, coverage, husbandry, body condition, nutrition and other animal factors; (2) vaccine potency at point of production; or (3) vaccine delivery (e.g. cold chain or shelf life adherence).

The reduction in VE with increasing time since vaccination was as expected, with protection due to the TUR 11 vaccine declining after 100 days. The Shamir VE appeared to decline sooner (after 50 days) (Table 2).

The findings differ to those from a PD_50_ challenge study. A high potency (>6PD_50_) Shamir vaccine held in the EU vaccine bank protected against clinical FMD when challenged with the Turkish FMD Asia-1 Sindh-08 field virus [15]. Differences in protection will partly reflect differences in potency as poor vaccine match may be overcome if high potency vaccines are used [16] and in the challenge study the vaccine used was likely to be much greater than 6PD_50_. Furthermore, in the challenge study, animals were assessed at time of peak immunity (21 days after vaccination), whereas in the VE study time between vaccination and challenge varied from one to five months.

NSP serology is a sensitive method of detecting animals with significant systemic viral replication [17]. As this will correlate with virus shedding, NSP status is a suitable outcome for vaccine evaluation. Şap institute vaccines are quality controlled for NSP purity, however, NSP sero-positivity following multiple vaccine doses can occur. Including age in the model helped control for this. NSP sero-status was considered together with Asia-1 SP sero-status to increase specificity. Cross-reactivity between SP antibodies of different serotypes could lead to falsely classifying animals with prior A or O infections as infected during the investigated Asia-1 outbreak, however, no recent prior outbreaks had occurred.

For twelve months after the loss of maternal immunity (ages 7–18 months) animals were particularly susceptible to FMD. As this age group are frequently traded, they should be targeted by control measures as a high risk group.

FMD is one of the most infectious animal pathogens with estimates for the basic reproduction number (*R*_0_) within a herd ranging from 2 to 70 [18]. Furthermore, husbandry practices mean that villages in Turkey can be considered a well-mixed population equivalent to a herd. According to herd-immunity theory [19], with 69% VE and coverage levels found during these investigations vaccination could suppress within-village outbreaks with an *R*_0_ < 1.4 for Afyon-1 (coverage = 42%) up to *R*_0_ < 2.25 for Denizli (coverage = 83%). With 100% coverage the vaccine could control an outbreak with *R*_0_ < 3.2. An inability to control outbreaks with FMD vaccines has been reported before [18].

Although there are limitations with this sort of calculation, it indicates that additional sanitary measures are required to reduce virus exposure and *R*_0_ to a level that will not overwhelm vaccine protection. Routine culling is not feasible in highly endemic regions leaving improved biosecurity, particularly isolation of infected and high risk premises, as the best option. Not surprisingly use of communal grazing was an important risk factor.

Although there is less contact between animals in adjacent villages, common grazing usually overlaps. With high attack rates (35% in TUR 11 vaccinated cattle) and large numbers of cattle per village (≥450 cattle), each infected village will contain >100 diseased cattle. When relying on vaccination alone, transmission by one or more infected animals to neighbouring villages or livestock markets seems likely.

## 5 Conclusion

In this study we found that the FMD Asia-1 TUR 11 vaccine provided reasonable protection against disease and infection with the homologous field virus. However, vaccine performance varied from farm to farm. Although the vaccine performed as expected for a standard potency FMD vaccine [13], widespread transmission still occurred, partly due to limited vaccine coverage. However, there is a mismatch between the very high vaccine effectiveness required to control FMD and the actual effectiveness of standard FMD vaccines. The use of other control measures in conjunction with vaccination will help to overcome this mismatch.

The FMD Asia-1 Shamir vaccine did not appear to protect in the outbreak investigated.

Vaccine effectiveness should be monitored, particularly when there are outbreaks within a vaccinated population.

## Conflict of interest statement

Dr A.N. Bulut is employed by the Şap institute, which manufactures the vaccines under evaluation.

## Figures and Tables

**Table 1:** Details of the four vaccine effectiveness studies performed in Turkey.

Investigation Investigation dates	No. of villages	Village livestock population	Husbandry	Management groups sampled	FMD Asia-1 vaccine	Time between vaccination & outbreak	Date outbreak started
Ardahan province 27–30 September 2011	6	450–3000 cattle 0–300 sheep	Extensive grazing	16	Shamir	62–152 days	19 June – 24 September 2011
Afyon province-1 9–14 January 2012	2	700–2300 cattle 0–4000 sheep	Always housed	19	TUR 11	48–65 days	20 & 22 November 2012
Denizli province 26 June – 1 July 2012	2	470–550 cattle 400–1000 sheep	Housed and grazing	75	TUR 11	39–126 days	26–29 May 2012
Afyon province-2 10–13 July 2012	2	2000 cattle 1000–1500 sheep	Housed and grazing	31	TUR 11	65–85 days	13 June 2012

**Table 2:** Descriptive statistics, categorical univariable association of risk factors with clinical FMD and stratum specific vaccine effectiveness for the TUR 11 vaccinated animals only except where indicated. Animals over four months only.

Variable	Category	% in each category	Cases/Total	Chi-squared *p*-value	Unvaccinated cattle Cases/Total	Vaccinated cattle Cases/Total		TUR 11 Vaccine effectiveness (95% CI) Unvaccinated taken from all studies
						TUR 11	Shamir	
Age (months) *n* = 1230	4–6	11%	90/132 (68%)	*p* < 0.001	27/47 (57%)	18/37 (49%)	45/48 (94%)	15% (−28% to 44%)
	7–18	31%	224/381 (59%)		74/111 (67%)	77/189 (41%)	73/81 (90%)	40% (15%–51%)
	18–36	33%	192/406 (47%)		74/127 (58%)	66/212 (33%)	52/77 (68%)	44% (28%–56%)
	>36	25%	108/311 (35%)		34/81 (42%)	53/176 (30%)	21/54 (39%)	28% (0%–49%)

Common grazing *n* = 1230	Yes	56%	405/693 (58%)	*p* = 0.02	52/73 (71%)	165/371 (44%)	188/249 (76%)	38% (22%–51%)
	No	44%	209/537 (39%)		157/293 (54%)	49/233 (21%)	3/11 (27%)	61% (50%–70%)

Breed *n* = 1100	Black & white	45%	228/496 (46%)	*p* < 0.001	93/139 (67%)	135/357 (38%)	–	44% (31%–53%)
	Continental	23%	123/250 (49%)		34/61 (56%)	14/42 (33%)	154/195 (79%)	27% (1%–46%)
	Local	32%	223/354 (63%)		55/117 (47%)	55/135 (41%)	34/54 (63%)	29% (−13% to 57%)

Sex *n* = 1229	Male	29%	207/352 (59%)	*p* < 0.001	101/145 (70%)	48/143 (34%)	58/64 (91%)	52% (39%–62%)
	Female	71%	406/877 (46%)		108/221 (49%)	166/461 (36%)	132/195 (68%)	26% (11%–39%)

Trimester (>14 months age only) *n* = 277	Not pregnant	44%	43/122 (35%)	*p* = 0.3	5/14 (36%)	38/108 (35%)	–	1% (−107% to 53%)
	First	25%	18/69 (26%)		6/14 (43%)	12/55 (23%)	–	49% (−11% to 77%)
	Second	16%	10/44 (23%)		2/4 (50%)	8/40 (20%)	–	60% (−27% to 87%)
	Third	15%	10/42 (24%)		0/3 (0%)	10/39 (26%)	–	–

Management group size (cattle) *n* = 1230	<11	12%	67/143 (47%)	*p* = 0.09	33/52 (63%)	31/80 (39%)	3/11 (27%)	39% (11%–58%)
	11–20	32%	193/396 (49%)		129/182 (71%)	64/214 (30%)		58% (48%–66%)
	21–30	8%	59/98 (62%)		7/27 (26%)	52/68 (76%)		−195% (−)
	>30	48%	295/596 (49%)		40/105 (38%)	67/242 (28%)	188/249 (76%)	27% (0%–47%)

Time between vaccination and outbreak *n* = 864	39–50 days	41%	130/339 (38%)	*p* < 0.0001	–	127/328 (39%)	3/11 (27%)	–
	51–100 days	43%	170/379 (45%)		–	80/267 (30%)	90/112 (80%)	–
	101–152 days	16%	105/146 (72%)		–	7/9 (78%)	98/137 (72%)	–

Herd vaccine coverage *n* = 1230	0	22%	144/270 (53%)	*p* = 0.02	144/270 (53%)	–	–	–
	1%–39%	5%	36/56 (64%)		31/45 (69%)	3/8 (38%)	2/3 (67%)	46% (−36% to 79%)
	40%–69%	4%	23/46 (50%)		11/18 (61%)	0/5 (0%)	12/23 (52%)	15% (−72% to 58%)
	70%–94%	14%	77/174 (44%)		21/29 (72%)	41/126 (33%)	15/19 (79%)	58% (39%–71%)
	>94%	56%	334/684 (49%)		2/4 (50%)	170/465 (37%)	162/215 (75%)	26% (−3% to 100%)

Investigation *n* = 1230	Ardahan	24%	207/296 (70%)	*p* < 0.001	19/47 (40%)	–	188/249 (76%)	−87% (−144% to −44%) Shamir
	Afyon-1	18%	78/218 (36%)		64/127 (50%)	11/80 (14%)	3/11 (27%)	73% (56%–84%)
	Denizli	33%	189/405 (47%)		55/68 (81%)	134/337 (40%)	–	51% (38%–61%)
	Afyon-2	25%	140/311 (45%)		71/124 (57%)	69/187 (37%)	–	36% (17%–50%)

	Total	100%	614/1230 (50%)		209/366 (57%)	214/604 (35%)	191/260 (73%)	–

**Table 3:** The risk of clinical FMD by number of doses of the FMD Asia-1 Shamir vaccine received in an animal's lifetime. All cattle were over four months old and number of vaccine doses is highly correlated with age. NB: in the TUR 11 vaccine investigations animals had only received a maximum of one dose as this was a new vaccine. Furthermore these cattle had never previously been vaccinated for FMD Asia-1.

Number of vaccine doses in lifetime	Incidence risk	Relative risk
	Cases/total	[95% CI]
0	14/21 (67%)	0.75 [0.59–0.96]
1	102/115 (89%)	Baseline
2–5	58/84 (69%)	0.78 [0.67–0.9]
≥6	18/45 (40%)	0.45 [0.35–0.58]

**Table 4:** Bayesian multivariable regression analysis of vaccine effectiveness against FMD from field studies conducted in Turkish villages. A complimentary log–log link function was used. Median parameter estimates are presented with credible intervals.

Risk factor		Vaccine effectiveness [95% Credible Interval]		
		FMD	Severe FMD^a^	FMD virus infection^b^
Recently vaccinated	TUR 11 vaccine	69% [50%–81%]	83% [67%–92%]	63% [29%–81%]
	Shamir vaccine	−36% [−137% to 22%]	−129% [−600% to 21%]	1% [−200% to 68%]

		Rate ratio		
Avoid common grazing		0.2 [0.1–0.36]	0.1 [0.03–0.27]	0.3 [0.1–0.7]
Every month over 15 months		0.98 [0.977–0.99]	0.99 [0.98–0.995]	1.03 [1.01–1.04]
Herd size >30		0.25 [0.1–0.5]	0.06 [0.01–0.23]	0.6 [0.2–1.5]
Standard deviation of random intercept	Herd-group	1.4^c^	2	2
	Village	6^c^	7	7
Deviance information criterion		1315	832	446
Chi-sq. goodness of fit test Bayesian *p*-value		0.2	0.1	0.1

a Stopped eating or oral lesions with combined diameter greater than 50% width of hard palate.

b Considered infected during the outbreak if FMD Asia-1 SP antibody titre >32 and positive for NSP antibodies.

c When standard deviations of the random intercept were converted from the complimentary log–log scale an unvaccinated individual, ≤15 months age, from a herd of ≤30 cattle at common grazing had a mean probability of developing FMD per day of between 0.1% and 40% for 95% of herds. Between villages this figure varied between 0% and 100% for 95% of villages.
