# Supplementary material for: Retrospective evaluation of foot-and-mouth disease vaccine effectiveness in Turkey
Source: Vaccine. 2014 Apr 1;32(16):1848–55. doi: 10.1016/j.vaccine.2014.01.071 (PMC3991324; doi:10.1016/j.vaccine.2014.01.071)
Supplement: Supplementary file 1 [file mmc1.docx]

Table S1: Information collected during retrospective outbreak investigation. A copy of the investigation questionnaire is available on request.

| Holding details:  *-Province, district, village and farmer name, what type of grazing used (none, private, common), herd size.* |
| --- |
| Animal details:  *-Animal ear tag number, age, sex, housing group, ear tag number of mother if <3 months, breed,* _a_*pregnant (yes or no),* _a_ *number of months gestation.* |
| Vaccination details:  *-Date of last vaccination, type and batch number of FMD vaccine received last,* _a_*number of vaccine doses received in lifetime, time between outbreak and last vaccination, group vaccine coverage at last vaccination round [calculated from data].* |

_a_ information not always available.

Table S2 (a): Stratified FMD incidence in vaccinated and unvaccinated cattle. Animals over four months only. Results are for Ardahan and Afyon-1 investigations.

|  |  | **Ardahan** | | | **Afyon-1** | | |
| --- | --- | --- | --- | --- | --- | --- | --- |
| **Variable** | **Category** | **Unvaccinated** Cases/Total | **Vaccinated**  Cases/Total | | **Unvaccinated** Cases/Total | **Vaccinated**  Cases/Total | |
|  |  |  | **TUR 11** | **Shamir** |  | **TUR 11** | **Shamir** |
| **Age**  **(months)**  **n=1230** | **4-6** | 15/26 (58%) | - | 45/48 (94%) | 1/1 (100%) | - | - |
|  | **7-18** | 3/5 (60%) | - | 71/78 (91%) | 23/39 (59%) | 6/36 (17%) | 2/3 (67%) |
|  | **18-36** | 1/16 (6%) | - | 52/76 (68%) | 29/50 (58%) | 3/27 (11%) | 0/1 (0%) |
|  | **>36** | - | - | 20/47 (43%) | 11/37 (30%) | 2/17 (12%) | 1/7 (14%) |
| **Common Grazing**  **n=1230** | **Yes** | 19/30 (63%) | - | 188/249 (76%) | - | - | - |
|  | **No** | 0/17 (0%) | - | - | 64/127 (50%) | 11/80 (14%) | 3/11 (27%) |
| **Breed**  **n=1100** | **Black & White** | - | - | - | - | - | - |
|  | **Continental** | 2/7 (29%) | - | 34/54 (63%) | 9/14 (64%) | - | - |
|  | **Local** | 154/195 (79%) | - | 17/40 (43%) | 28/64 (44%) | 1/10 (10%) | - |
| **Sex**  **n=1229** | **Male** | 15/22 (68%) | - | 57/62 (92%) | 29/44 (66%) | 2/30 (7%) | 1/2 (50%) |
|  | **Female** | 4/25 (16%) | - | 130/186 (70%) | 35/83 (42%) | 9/50 (18%) | 2/9 (22%) |
| **Management group size**  **(cattle)**  **n=1230** | **<11** | - | - | - | 19/33 (58%) | 5/24 (21%) | 3/11 (27%) |
|  | **11-20** | - | - | - | 38/67 (57%) | 6/56 (11%) | - |
|  | **21-30** | - | - | - | 7/27 (26%) | - | - |
|  | **>30** | 19/47 (40%) | - | 188/249 (76%) | - | - | - |
| **Time between vaccination and outbreak** | **39-50 days** | - | - | - | 12/15 (80%) | - | 3/11 (27%) |
|  | **51-100 days** | 1/1 (100%) | - | 90/112 (80%) | 52/112 (46%) | 11/80 (14%) | - |
|  | **101-152 days** | 18/46 (39%) | - | 98/137 (72%) | - | - | - |
| **Herd vaccine coverage**  **n=1230** | **0** | 2/24 (8%) | - | - | 34/80 (43%) | - | - |
|  | **1% - 39%** | - | - | - | 25/36 (69%) | 2/3 (67%) | 2/3 (67%) |
|  | **40% - 69%** | 11/15 (73%) | - | 12/23 (52%) | 0/3 (0%) | 0/5 (0%) | - |
|  | **70% - 94%** | 4/4 (100%) | - | 15/19 (79%) | 5/8 (63%) | 5/35 (14%) | - |
|  | **>94%** | 2/4 (50%) | - | 161/207 (78%) | - | 4/37 (11%) | 1/8 (13%) |

Table S2 (b): Stratified FMD incidence in vaccinated and unvaccinated cattle. Animals over four months only. Results are stratified for Denizli and Afyon-2 investigations.

|  |  | **Denizli** | | **Afyon-2** | |
| --- | --- | --- | --- | --- | --- |
| **Variable** | **Category** | **Unvaccinated** Cases/Total | **Vaccinated**  Cases /Total | **Unvaccinated**  Cases/Total | **Vaccinated**  Cases/Total |
|  |  |  | **TUR 11** |  | **TUR 11** |
| **Age**  **(months)**  **n=1230** | **4-6** | 3/6 (50%) | 14/32 (44%) | 8/14 (57%) | 4/5 (80%) |
|  | **7-18** | 20/24 (83%) | 45/111 (41%) | 28/43 (65%) | 26/42 (62%) |
|  | **18-36** | 18/19 (95%) | 28/58 (48%) | 26/42 (62%) | 35/117 (30%) |
|  | **>36** | 14/19 (74%) | 47/136 (35%) | 9/25 (36%) | 4/23 (17%) |
| **Common Grazing**  **n=1230** | **Yes** | 46/57 (81%) | 33/135 (24%) | 24/32 (75%) | 64/169 (38%) |
|  | **No** | 9/11 (82%) | 101/202 (50%) | 47/92 (51%) | 5/18 (28%) |
| **Breed**  **n=1100** | **Black & White** | - | - | - | - |
|  | **Continental** | 55/68 (81%) | 134/237 (40%) | 61/111 (55%) | 56/155 (36%) |
|  | **Local** | - | - | 10/13 (77%) | 13/32 (41%) |
| **Sex**  **n=1229** | **Male** | 13/19 (68%) | 22/55 (40%) | 44/60 (73%) | 24/58 (41%) |
|  | **Female** | 42/49 (86%) | 112/282 (40%) | 27/64 (42%) | 45/129 (35%) |
| **Management group size**  **(cattle)**  **n=1230** | **<11** | - | 26/55 (47%) | 14/19 (74%) | 0/1 (0%) |
|  | **11-20** | 55/78 (80%) | 45/124 (36%) | 36/47 (77%) | 13/34 (38%) |
|  | **21-30** | - | 52/68 (76%) | - | - |
|  | **>30** | - | 11/90 (12%) | 21/58 (36%) | 56/152 (37%) |
| **Time between vaccination and outbreak** | **39-50 days** | 9/12 (75%) | 127/328 (39%) | - | - |
|  | **51-100 days** | - | - | 71/124 (57%) | 69/187 (37%) |
|  | **101-152 days** | 46/56 (82%) | 7/9 (78%) | - | - |
| **Herd vaccine coverage**  **n=1230** | **0** | 54/66 (81%) | - | 54/100 (54%) | - |
|  | **1% - 39%** | - | - | 6/9 (67%) | 1/5 (20%) |
|  | **40% - 69%** | 1/2 (50%) | 9/20 (45%) | - | - |
|  | **70% - 94%** | - | 125/317 (39%) | 11/15 (73%) | 27/71 (38%) |
|  | **>94%** | - | - | - | 41/111 (37%) |

Table S3: Crude vaccine effectiveness against various FMD outcome measures [both Shamir and TUR 11 vaccines].

| **Category** | **Cases/Total (%)** | | | **Vaccine effectiveness**  **(95% CI)**  **TUR 11** | **Vaccine effectiveness**  **(95% CI)**  **Shamir** |
| --- | --- | --- | --- | --- | --- |
|  | **Unvaccinated** | **Vaccinated TUR 11** | **Vaccinated Shamir** |  |  |
| FMD seen by farmer | 167/358 (47%) | 150/604 (25%) | 55/210 (74%) | 47% (37% to 56%) | -58% (-83% to -37%) |
| FMD seen on clinical exam | 142/262 (54%) | 138/352 (39%) | 145/204 (71%) | 28% (14% to 40%) | -31% (-52% to -13%) |
| Stopped eating | 113/366 (31%) | 118/604 (20%) | - | 37% (21% to 50%) | - |
| Severe disease* | 115/366 (31%) | 122/604 (20%) | 35/260 (13%) | 36% (20% to 495) | 57% (40% to 69%) |

*Stopped eating or oral lesions with combined diameter greater than 50% width of hard palate.

**Table S4: Incidence and vaccine effectiveness based on serology. Structural protein antibodies (SP) and non-structural proteins antibodies (NSP).**

| **Serological criteria** | **Cases/Total (%)** | | | **Vaccine effectiveness**  **(95% CI)**  **TUR 11** | **Vaccine effectiveness**  **(95% CI)**  **Shamir** |
| --- | --- | --- | --- | --- | --- |
|  | **Unvaccinated** | **Vaccinated TUR 11** | **Vaccinated Shamir** |  |  |
| NSP positive | 228/259 (88%) | 213/327 (65%) | 189/205 (92%) | 26% (19% to 33%) | -5% (-13% to 2%) |
| Asia-1 SP Titre ≥32 | 235/257 (90%) | 297/327 (91%) | 139/145 (92%) | 1% (-18% to 17%) | -5% (-12% to 2%) |
| Asia-1 SP Titre ≥256 | 172/257 (67%) | 163/327 (50%) | 133/145 (92%) | 26% (14% to 36%) | -37% (-53% to -22%) |
| NSP positive & Asia-1 SP Titre>32 | 222/257 (86%) | 211/327 (65%) | 129/145 (89%) | 25% (17% to 32%) | -4% (-14% to 5%) |
| NSP positive & Asia-1 SP Titre>256 | 169/257 (66%) | 157/327 (48%) | 129/145 (89%) | 27% (15% to 37%) | -34% (-51% to -19%) |
